# Inhibition of the PINK1-Parkin Pathway Enhances the Lethality of Sorafenib and Regorafenib in Hepatocellular Carcinoma

**DOI:** 10.3389/fphar.2022.851832

**Published:** 2022-03-15

**Authors:** Shun Zhang, Yixin Wang, Yifan Cao, Jin Wu, Zubin Zhang, Haigang Ren, Xiaohui Xu, Elena Kaznacheyeva, Qing Li, Guanghui Wang

**Affiliations:** ^1^ Laboratory of Molecular Neuropathology, Jiangsu Key Laboratory of Neuropsychiatric Diseases and College of Pharmaceutical Sciences, Soochow University, Suzhou, China; ^2^ Department of General Surgery, the First People’s Hospital of Taicang, Taicang Affiliated Hospital of Soochow University, Suzhou, China; ^3^ Institute of Cytology RAS, Saint-Petersburg, Russia; ^4^ Department of Gastroenterology, the First People’s Hospital of Taicang, Taicang Affiliated Hospital of Soochow University, Suzhou, China; ^5^ Center of Translational Medicine, the First People’s Hospital of Taicang, Taicang Affiliated Hospital of Soochow University, Suzhou, China

**Keywords:** HCC, sorafenib, regorafenib, MPTP, PINK1, mitophagy, mitofission

## Abstract

Hepatocellular carcinoma (HCC) is one of the most common fatal malignancies and the main cause of cancer-related deaths. The multitarget tyrosine kinase inhibitors (TKIs) sorafenib and regorafenib are systemic therapeutic drugs approved for the treatment of HCC. Here, we found that sorafenib and regorafenib injured mitochondria by inducing mitochondrial Ca^2+^ (mtCa^2+^) overload and mitochondrial permeability transition pore (mPTP) opening, resulting in mitochondria-mediated cell death, which was alleviated by cyclosporin A (CsA), an inhibitor of mPTP. Meanwhile, mPTP opening caused PINK1 accumulation on damaged mitochondria, which recruited Parkin to mitochondria to induce mitophagy. Inhibition of autophagy by the lysosomal inhibitor chloroquine (CQ) or inhibition of mitochondrial fission by mdivi-1 aggravated sorafenib- and regorafenib-induced cell death. Moreover, knockdown of PINK1 also promotes sorafenib- and regorafenib-induced cell death. An *in vivo* study showed that sorafenib and regorafenib inhibited HepG2 cell growth more effectively in PINK1 knockdown cells than in shNTC cells in null mice. Thus, our data demonstrate that PINK1-Parkin-mediated mitophagy alleviates sorafenib and regorafenib antitumor effects *in vitro* and *in vivo*.

## Introduction

Hepatocellular carcinioma (HCC) is the third leading cause of cancer mortality worldwide (Sung et al., 2021). Although different kinds of therapeutic schedules have been applied to patients, most of them still need systematic therapy. The multitarget tyrosine kinase inhibitor (TKI) sorafenib is approved for the first-line systematic therapy of advanced HCC by the FDA, which expands patient median survival from 7.9 to 10.7 months (Abou-Alfa et al., 2006; Llovet et al., 2008; Cheng et al., 2009). However, the therapeutic effects of sorafenib are transitory, and most patients develop disease progression after treatment for 4–5 months (Llovet et al., 2008). Regorafenib is found to improve the survival of patients who tolerate but progress on sorafenib and is approved as a second-line therapy (Llovet et al., 2008; Bruix et al., 2017). Given their similar structures, sorafenib and regorafenib both inhibit a variety of kinase activities, including vascular endothelial growth factor receptor (VEGFR), platelet-derived growth factor receptor (PDGFR) and Raf-1 proto-oncogene (RAF), to block cell proliferation and angiogenesis.

Mitochondria are the center of energy metabolism in cells. Apart from the well-known Warburg effect, mitochondria play an important role in cancer growth, proliferation, progression, and tumor metastasis (Vyas et al., 2016). Many therapeutic approaches that target different functions of mitochondria, such as mitochondrial metabolism (Krall et al., 2021), mitochondrial apoptosis (Cheng et al., 2020) and mitochondrial antigen presentation (Pierini et al., 2015), have been exploited in cancer treatment. It has been well documented that sorafenib and regorafenib inhibit mitochondrial respiratory chain complexes and induce mitochondrial dysfunction and mitochondria-dependent apoptosis by regulating BH3-only proteins (Fernando et al., 2012; Chen et al., 2014; Zhang et al., 2017). In contrast to the induction of cell death by mitochondria when cells are treated with antitumor agents, mitochondria in tumors often make accordingly changes to adapt or resist treatment by switching from glycolysis to OXPHOS (Trotta et al., 2017) or increasing mitochondrial fission and mitophagy (Lin et al., 2020). However, the effects of mitophagy in response to sorafenib and regorafenib are still unclear.

Mitochondria are dynamic organelles. Once injured, dynamin-related protein 1 (Drp1) is activated and translocated to mitochondria to separate the dysfunctional parts through mitochondrial fission (Jin et al., 2021). Segregated mitochondria are degraded by mitophagy, which is a selective form of autophagy that maintains mitochondrial homeostasis by eliminating damaged or dysfunctional mitochondria, hence protecting cells from death (Ma et al., 2020; Gao et al., 2021). The PTEN-induced putative kinase 1 (PINK1)-Parkin pathway is the most reported pathway in mitophagy induction. Upon loss of mitochondrial membrane potential (ΔΨm), the N-terminus of PINK1 fails to be transported into the inner mitochondrial membrane (IMM), where PINK1 is processed by several proteases (Harper et al., 2018; Palikaras et al., 2018). As a consequence, PINK1 stabilizes at the outer mitochondrial membrane (OMM), recruiting and activating Parkin (Ge et al., 2020; Antico et al., 2021), an E3 ligase that ubiquitinates OMM proteins, which leads to the recognition of ubiquitinated mitochondria by autophagic receptors and the engulfment of mitochondria by autophagosomes for degradation.

Here, we showed that sorafenib and regorafenib induce mitochondrial Ca^2+^ (mtCa^2+^)-mediated mitochondrial permeability transition pore (mPTP) opening, which leads to loss of ΔΨm, thereby triggering mitochondrial fission and PINK1/Parkin-mediated mitophagy. Blockage of mitophagy sensitizes HCC cells to sorafenib- and regorafenib-induced cell death *in vivo* and *in vitro*.

## Materials and Methods

### Cell Culture and Drug Treatment

HepG2, Hep3B and HEK293 cells were cultured in Dulbecco’s modified Eagle’s medium (Gibco, Los Angeles, CA) containing 10% fetal bovine serum (FBS, Gibco) supplemented with penicillin (100 U/ml) and streptomycin (100 μg/ml; Gibco). Sorafenib, regorafenib and CsA were purchased from MedChemExpress (Monmouth Junction, NJ, United States) and dissolved in dimethylsulfoxide (DMSO). Chloroquine was purchased from MedChemExpress and dissolved in PBS. BAPTA-AM was purchased from Selleck (Houston, TX, United States). Bafilomycin A1 were purchased from Sigma (St. Louis, MO, United States).

### Tumor Xenografts in Nude Mice

All mouse experiments were carried out according to the institutional guidelines for the use and care of animals, and all procedures were approved by the ethical committee of Soochow University. HepG2-shCTR and HepG2-shPINK1 cells (2 × 10^6^) were resuspended in 100 µL PBS and injected subcutaneously into 6-week-old BALB/c nude mice (Shanghai SLAC Laboratory Animal Co., Ltd.). Tumor volumes were measured using an electronic caliper and calculated using the following formula: volume (cm^3^) = L × W^2^ × 0.5. Drug administration began when the tumors reached 200 mm^3^. Sorafenib (10 mg/kg/2 days) or regorafenib (10 mg/kg/2 days) was intraperitoneally injected for 20 days. Tumor volumes were measured every other day. At the end of the experiments, mice were sacrificed, and the tumors were removed, weighed and photographed.

### Western Blotting

Cells were lysed in 1 × SDS lysis buffer [50 mmol/L Tris-HCl (pH 7.5), 150 mmol/L NaCl, 1% Nonidet P40, and 0.5% sodium deoxycholate] supplemented with a protease inhibitor cocktail (Roche, Basel, Switzerland). Approximately 20 µg of cell lysate was isolated by SDS–PAGE and transferred onto a PVDF membrane (Millipore, Billerica, MA, United States). After blocking, the membranes were incubated with the following primary antibodies: anti-PINK1, anti-PARP, anti-cleaved caspase-9, and anti-phospho-Drp1 (Ser637) antibodies (Cell Signaling Technology, Danvers, MA, United States); anti-Tim23, anti-COXIV (Proteintech, Wuhan, China), anti-LC3, and anti-ubiquitination antibodies (Abclonal, Wuhan, China); anti-β-actin antibody (Sigma), anti-GFP, anti-Drp1 antibodies (Santa Cruz Biotechnology, Santa Cruz, CA, United States), and anti-α-tubulin antibody (Abcam, Cambridge, UK). The secondary antibodies, sheep anti-rabbit and anti-mouse IgG-HRP, were obtained from Thermo Fisher (Waltham, MA, United States). The proteins were visualized using an ECL detection kit (Thermo Fisher).

### Subcellular Fractionation Assay

HepG2 cells were treated with sorafenib or regorafenib for 12 h. The cells were then harvested, and the cytosolic and mitochondrial fractions were isolated using a mitochondrial isolation kit (Beyotime, Shanghai, China). Tim23 and α-tubulin were used as markers for mitochondria and the cytosol, respectively.

### Mitochondrial Membrane Potential Measurement

To measure ΔΨm, HepG2 cells were treated with 100 nmol/L TMRM (Thermo Fisher) for 15 min at 37°C after treatment with sorafenib or regorafenib. Healthy cells with ΔΨm are labeled with TMRM and present red fluorescence, while damaged cells with decreased ΔΨm exhibit decreased TMRM labeling (Yu et al., 2016). After incubation, the HepG2 cells were washed with PBS. Finally, the cells were imaged with an inverted IX71 microscope system (Olympus, Tokyo, Japan).

### Small Interfering RNA

RNA oligonucleotides were transfected into cells as described previously (Wang R. et al., 2019). Briefly, a mixture of Opti-MEM, RNAiMAX (Invitrogen, Carlsbad, CA, United States) and RNA oligonucleotides was incubated for 20 min at room temperature. The mixtures were then transfected into cells for 24 h. The cells were collected 72 h after transfection for further analysis. Oligonucleotides targeting human PINK1 were obtained from GenePharma (Shanghai, China). The sequences were as follows: si-PINK1 #1 sense 5′-CGC​UGU​UCC​UCG​UUA​UGA​ATT-3′ and anti-sense 5′-TTC​CTU​CCG​UGG​GAC​TT-3′.

### Cytotoxicity Assays

The cytotoxicity of LDH release was measured using a CytoTox 96 Non-Radioactive Cytotoxicity Assay (Promega, Madison, WI, United States). Briefly, 50 µL of growth medium was mixed with 50 µL of CellTiter-Glo and shaken for 20 min at room temperature. Luminescence was measured to detect cell cytotoxicity.

### Cell Viability Assays

Cells were seeded in 96-well plates and cultured overnight. After treatment with sorafenib and regorafenib for the indicated time, a 10 µL solution of cell counting kit-8 (CCK-8) was added to each well and incubated for 1 h. The absorbance was measured at 450 nm to calculate cell viability.

### Immunofluorescence

Immunofluorescence was performed as described previously (Guo et al., 2019). Cells were incubated with 4% paraformaldehyde for 10 min at room temperature and then permeabilized with 0.25% Triton X-100 for 10 min. Then, 1% bovine serum albumin was used for blocking for 1 h. The primary antibodies were incubated overnight at 4°C. Anti-Tom20 (Proteintech) antibody was used as the primary antibody. After incubation with the primary antibody, the cells were washed three times with PBS and then incubated with rhodamine (red)-conjugated secondary antibodies (Invitrogen, Carlsbad, CA, United States) for 1 h at room temperature. Finally, the cells were stained with DAPI for 10 min and imaged using an inverted IX71 microscope system (Olympus).

### Immunoprecipitation Assay

HepG2 cells were lysed with lysis buffer containing a protease inhibitor (Roche) on ice. The lysates were then sonicated and centrifuged to collect supernatants. After protein G agarose (Roche) was coupled with the indicated antibody, the supernatants were incubated with protein G agarose overnight at 4°C. The protein complexes coupled to Protein G Agarose were washed three times with lysis buffer. The immunoprecipitants and the input, which was 10% of total cell lysates, were analyzed using western blotting.

### Lentiviral Transduction

To obtain stable PINK1 knockdown cells, HepG2 cells were infected with lentivirus containing either control shRNA lentiviral particles (shNTC) or PINK1 shRNA lentiviral particles (shPINK1) (Shanghai Genechem Co., Ltd., Shanghai, China). The infected cells were subjected to G418 selection for 2 weeks.

### Statistical Analysis

The blots were quantified using Photoshop 7.0 (Adobe, San Jose, CA, United States), and the data were analyzed using GraphPad Prism 8.00 (GraphPad Software, Version X; La Jolla, CA, United States). Significant differences were evaluated using a two-tailed unpaired *t* test or one-way analysis of variance (ANOVA) followed by Dunnett’s multiple-comparisons test or two-way ANOVA followed by Tukey’s multiple-comparisons test. The criterion of significance was set at *p* < 0.05. The values are shown as the mean ± SEM.

## Results

### Sorafenib and Regorafenib Induce Mitochondria-Mediated Cell Death in HepG2 Cells by mtCa^2+^ Overload-Mediated mPTP Opening

**FIGURE 1:**
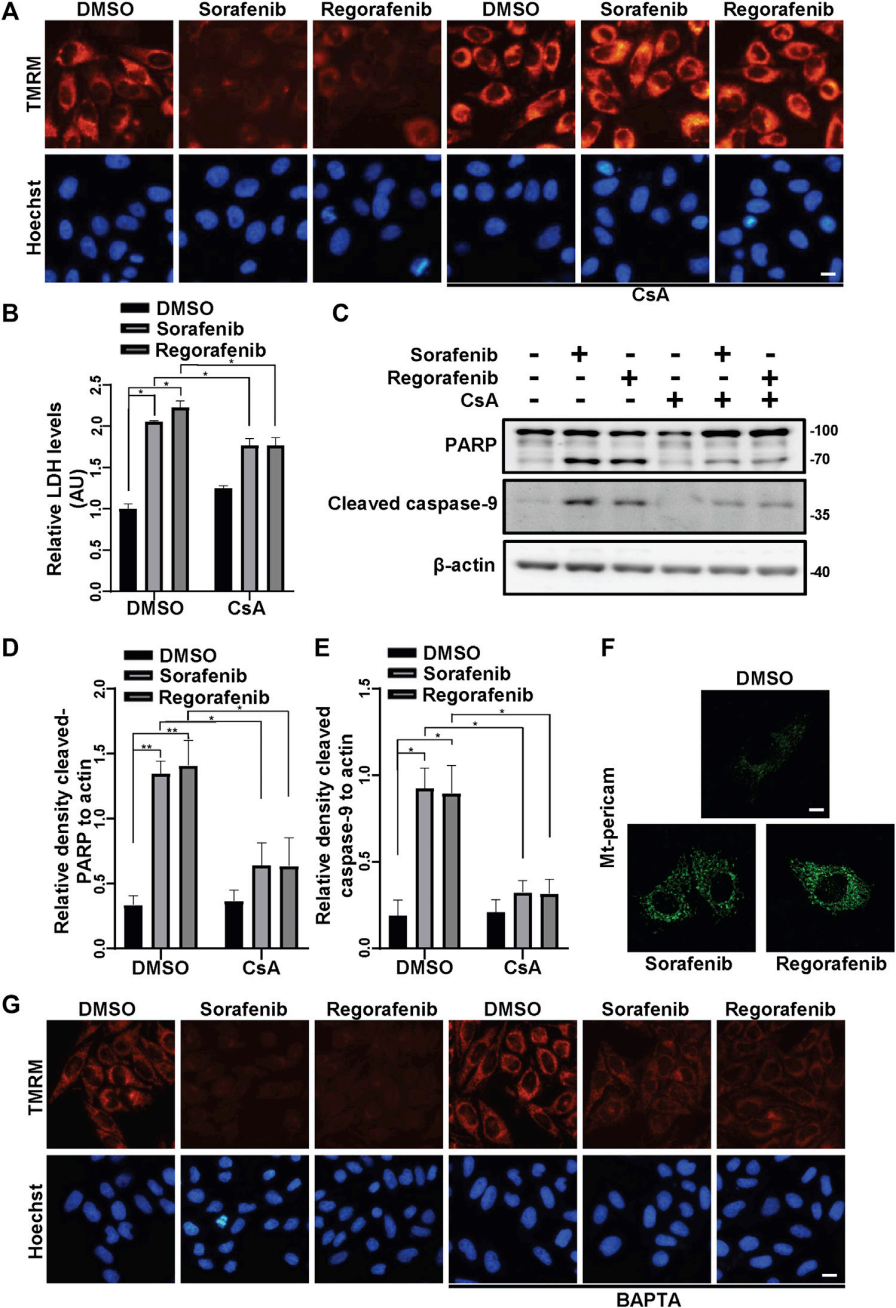
Sorafenib and regorafenib induce mitochondria-related cell death in HepG2 cells due to mtCa^2+^ overload-mediated mPTP opening. **(A)** HepG2 cells were pretreated with CsA (20 μM) for 1 h and treated with sorafenib (20 μM) or regorafenib (30 μM) for 4 h. The cells were then stained with TMRM to show the ΔΨm. Scale bar, 10 μm. **(B)** Cytotoxicity was measured using LDH in HepG2 cells pretreated with CsA (20 μM) for 1 h and treated with sorafenib (20 μM) or regorafenib (30 μM) for 24 h. Values are the mean ± SEM from four independent experiments (*p < 0.05, two-way ANOVA and Tukey’s multiple comparisons test). **(C)** Cleaved PARP and cleaved caspase-9 in HepG2 cells that were treated as in B are shown. **(D,E)** the relative densities of cleaved PARP **(D)** and cleaved caspase-9 **(E)** were quantified, and their protein levels were normalized to the loading control β-actin. Values are the mean ± SEM from three independent experiments (*p < 0.05, **p < 0.01, two-way ANOVA and Tukey’s multiple comparisons test). **(F)** HepG2 cells were transfected with mito-pericam and treated with sorafenib or regorafenib for 2 h (scale bar, 10 μm). **(G)** HepG2 cells were pretreated with BAPTA (20 μM) for 1 h and treated with sorafenib (20 μM) or regorafenib (30 μM) for 4 h. The cells were then stained with TMRM to show the ΔΨm. Scale bar, 10 μm.

Consistent with the findings by other investigators (Paech et al., 2018; Li et al., 2021), we observed that sorafenib and regorafenib induced the collapse of ΔΨm (Figure 1A). To further determine by which sorafenib and regorafenib induce mitochondrial dysfunction, we examined the effects of cyclosporin A (CsA), an inhibitor of mPTP, to determine whether mPTP is involved in the collapse of ΔΨm. In HepG2 cells that were treated with sorafenib and regorafenib, the loss of ΔΨm was observed (Figure 1A). However, in HepG2 cells that were treated with CsA, the loss of ΔΨm that was induced by sorafenib and regorafenib was blocked by CsA (Figure 1A). As loss of ΔΨm and opening of mPTP potentially induce mitochondria-mediated cell death, we performed LDH assays to examine cell viability. In HepG2 cells, blockade of mPTP opening with CsA suppressed sorafenib- and regorafenib-induced LDH release (Figure 1B). Moreover, CsA also decreased the cleavage of PARP (Figures 1C,D) and caspase-9 (Figures 1C,E), which was induced by sorafenib and regorafenib. mtCa^2+^ overload is one of the causative factors that induces mPTP opening. We therefore detected mtCa^2+^ in HepG2 cells that were transfected with mito-pericam, a Ca^2+^-sensitive fluorescent protein (Nagai et al., 2001; Filippin et al., 2005). Sorafenib and regorafenib enhanced the fluorescence intensity (Figure 1F), suggesting an overload of mtCa^2+^. In HepG2 cells that were treated with sorafenib/regorafenib, the calcium chelating agent BAPTA partially restored the ΔΨm (Figure 1G).

### Sorafenib and Regorafenib Activate the PINK1/Parkin Pathway *via* mPTP

**FIGURE 2:**
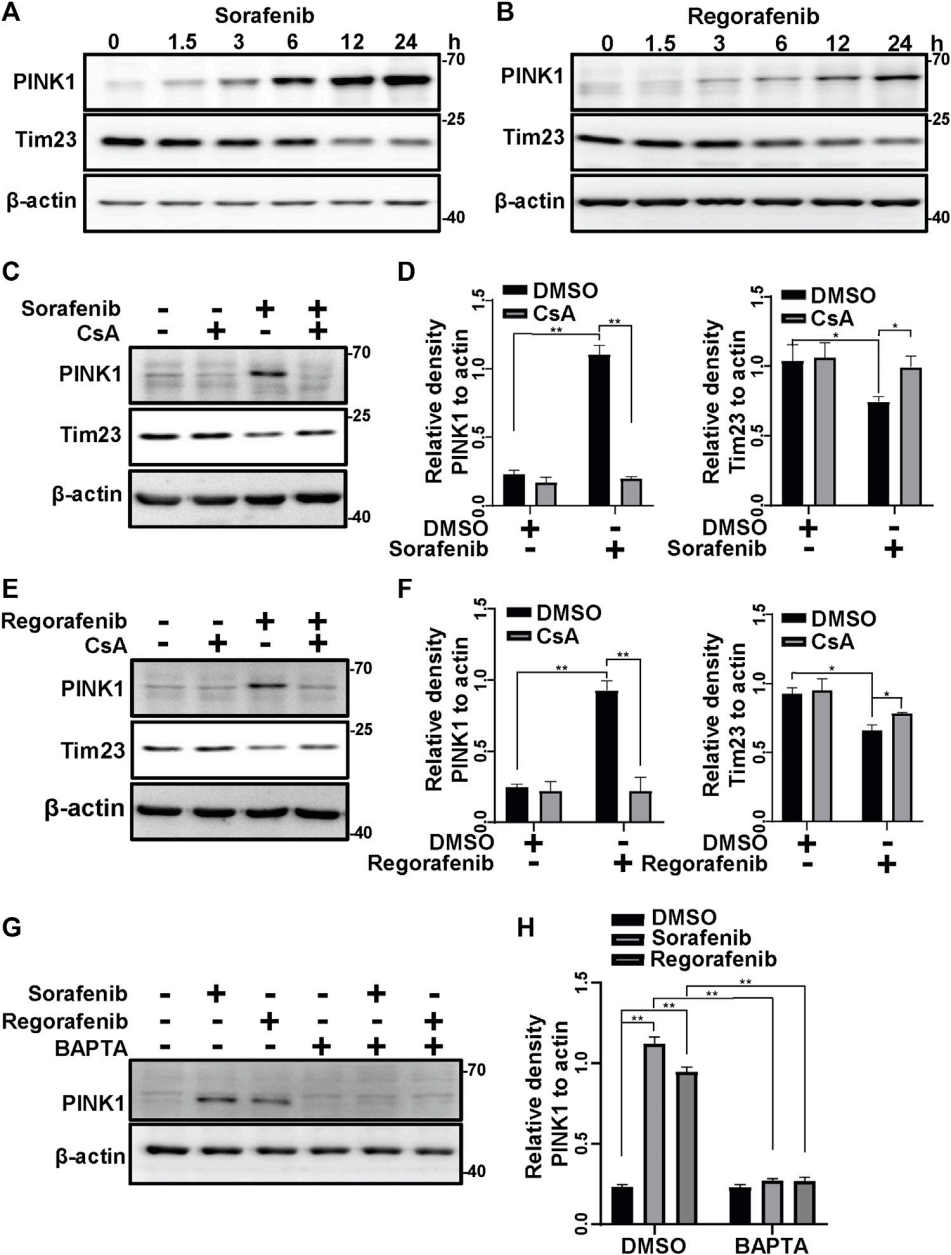
Sorafenib and regorafenib activate PINK1 *via* mPTP opening. **(A, B)** PINK1 and Tim23 in HepG2 cells that were treated with sorafenib (20 μM) or regorafenib (30 μM) at different time points (1.5, 3, 6, 12, 24 h) were labeled with the indicated antibodies. **(C)** HepG2 cells that were pretreated with CsA (20 μM) for 1 h and then treated with sorafenib (20 μM) for 4 h were subjected to western blotting with the indicated antibodies. **(D)** The relative densities of PINK1 and Tim23 were quantified, and their protein levels were normalized to the loading control β-actin. Values are the mean ± SEM from three independent experiments (*p < 0.05, **p < 0.01, two-way ANOVA and Tukey’s multiple comparisons test). **(E)** The experiments as same as **(C)** were performed but the cells were treated with regorafenib (30 μM) for 4 h. **(F)** The quantification of the data from **(E)** were analyzed as **(D)**. Values are the mean ± SEM from three independent experiments (*p < 0.05, **p < 0.01, two-way ANOVA and Tukey’s multiple comparisons test). **(G)** HepG2 cells that were pretreated with BAPTA (10 μM) for 1 h and treated with sorafenib (20 μM) or regorafenib (30 μM) for 4 h were subjected to western blotting with the indicated antibodies. **(H)** The relative density of PINK1 was quantified, and the protein level was normalized to that of the loading controls (β-actin). Values are the mean ± SEM from three independent experiments (**p < 0.01, two-way ANOVA and Tukey’s multiple comparisons test).

It is well known that loss of ΔΨm induces PINK1 accumulation on mitochondria (Jin et al., 2010). As sorafenib and regorafenib induced the collapse of ΔΨm (Figure 1A), we wondered whether PINK1 accumulated after sorafenib and regorafenib treatment. In HepG2 cells that were treated with sorafenib or regorafenib, PINK1 accumulated upon treatment with sorafenib (Figure 2A) or regorafenib (Figure 2B), which was accompanied by decreases in mitochondrial Tim23 levels (Figures 2A,B). Interestingly, PINK1 no longer accumulated, and Tim23 levels were not changed after CsA treatment in HepG2 cells that were treated with sorafenib (Figures 2C,D). Similar results were obtained in cells that were treated with CsA and regorafenib (Figures 2E,F). Furthermore, BAPTA also decreased the accumulation of PINK1 (Figures 2G,H).

**FIGURE 3:**
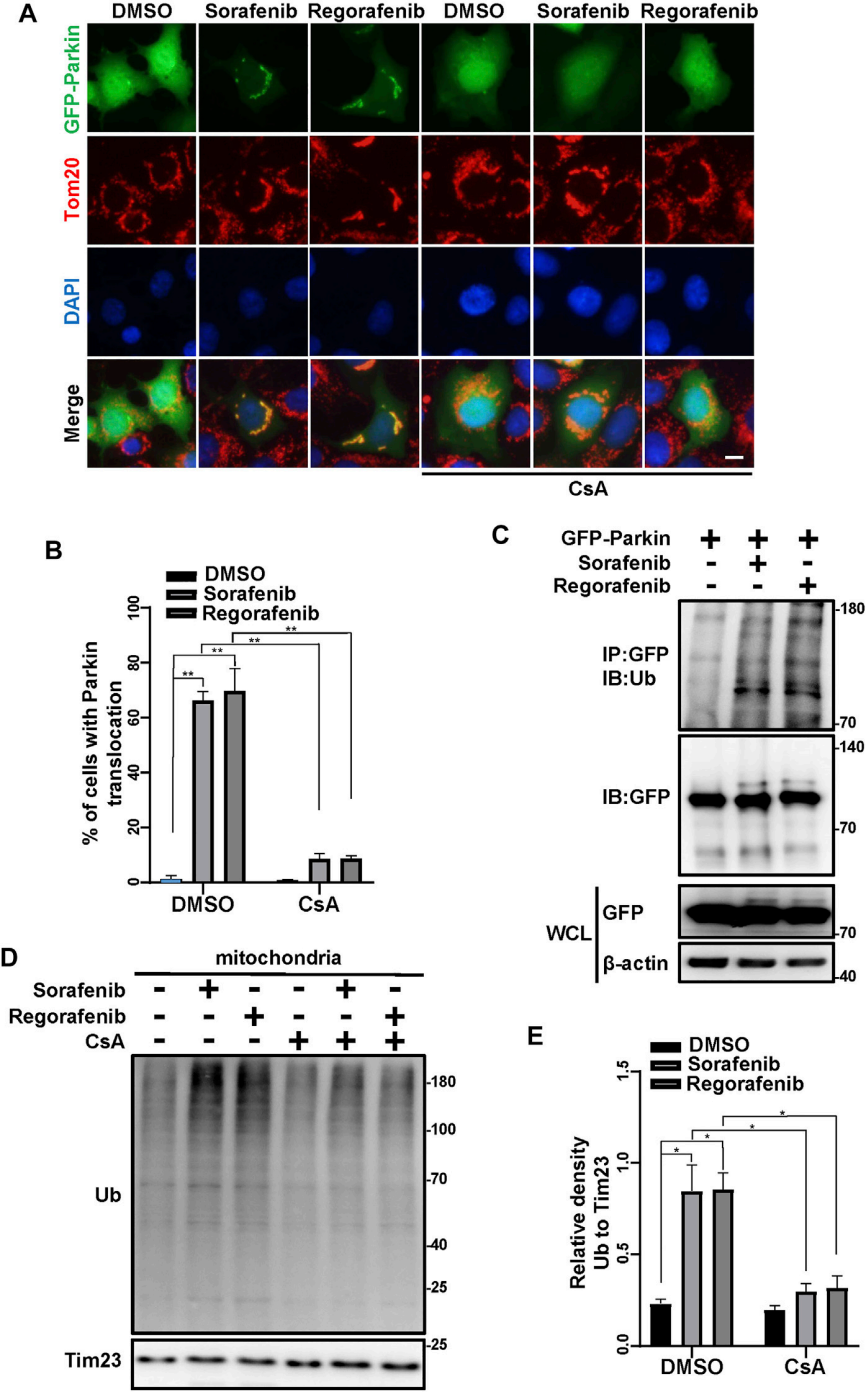
Sorafenib and regorafenib activate Parkin *via* mPTP opening. **(A)** HEK293 cells were transfected with EGFP-Parkin for 24 h. After pretreatment with CsA (20 μM) for 1 h, the cells were then treated with sorafenib (20 μM) or regorafenib (30 μM) for 4 h. Immunofluorescence of EGFP-Parkin is shown (blue, DAPI; green, EGFP-Parkin; red, Tom20; scale bar, 10 μm). **(B)** The density of cells with GFP-Parkin translocation as in A was quantified. Values are the mean ± SEM from three independent experiments (**p < 0.01, two-way ANOVA and Tukey’s multiple comparisons test). **(C)** Western blots showing Parkin ubiquitination in HepG2 cells that were transfected with EGFP-Parkin for 24 h and then treated with sorafenib or regorafenib for 4 h. The cell lysates were subjected to immunoprecipitation assays and western blotting with the indicated antibodies. **(D)** HepG2 cells were pretreated with CsA (20 μM) for 1 h or not and then treated with sorafenib (20 μM) or regorafenib (30 μM) for 4 h. The mitochondrial fractions were separated using a mitochondrial isolation kit. The ubiquitinated protein levels in the mitochondria were detected using western blotting. **(E)** The relative densities of ubiquitination were quantified, with the protein level normalized to the loading controls (Tim23). Values are the mean ± SEM from three independent experiments (*p < 0.05, two-way ANOVA and Tukey’s multiple comparisons test).

As sorafenib and regorafenib induce PINK1 accumulation (Figures 2A,B), we wondered whether Parkin is subsequently activated and translocated to mitochondria. In cells that were treated with sorafenib and regorafenib, Parkin was translocated to mitochondria (Figures 3A,B). However, the translocation of Parkin from the cytosol to mitochondria induced by sorafenib and regorafenib was blocked after treatment with CsA (Figures 3A,B). Moreover, PINK1 accumulation activates Parkin, leading to self-ubiquitination of Parkin (Geisler et al., 2010; Liu et al., 2021). In HepG2 cells that were treated with sorafenib and regorafenib, Parkin was more ubiquitinated than in HepG2 cells without treatment (Figure 3C). The accumulation of PINK1-induced mitochondrial translocation of Parkin leads to activation of Parkin, which ubiquitinates mitochondrial outer membrane proteins to induce mitophagy (Gao et al., 2015; Bingol and Sheng, 2016; Palikaras et al., 2018; Pickles et al., 2018). In isolated mitochondria from the cells that were treated with sorafenib or regorafenib, with or without CsA treatment, we observed that sorafenib and regorafenib induced polyubiquitination of mitochondrial proteins; however, CsA decreased sorafenib- and regorafenib-induced mitochondrial ubiquitination (Figures 3D,E). Thus, data suggest that blockade of mPTP by CsA decreases PINK1 accumulation and Parkin translocation to mitochondria.

### Sorafenib and Regorafenib Induce Mitophagy

**FIGURE 4:**
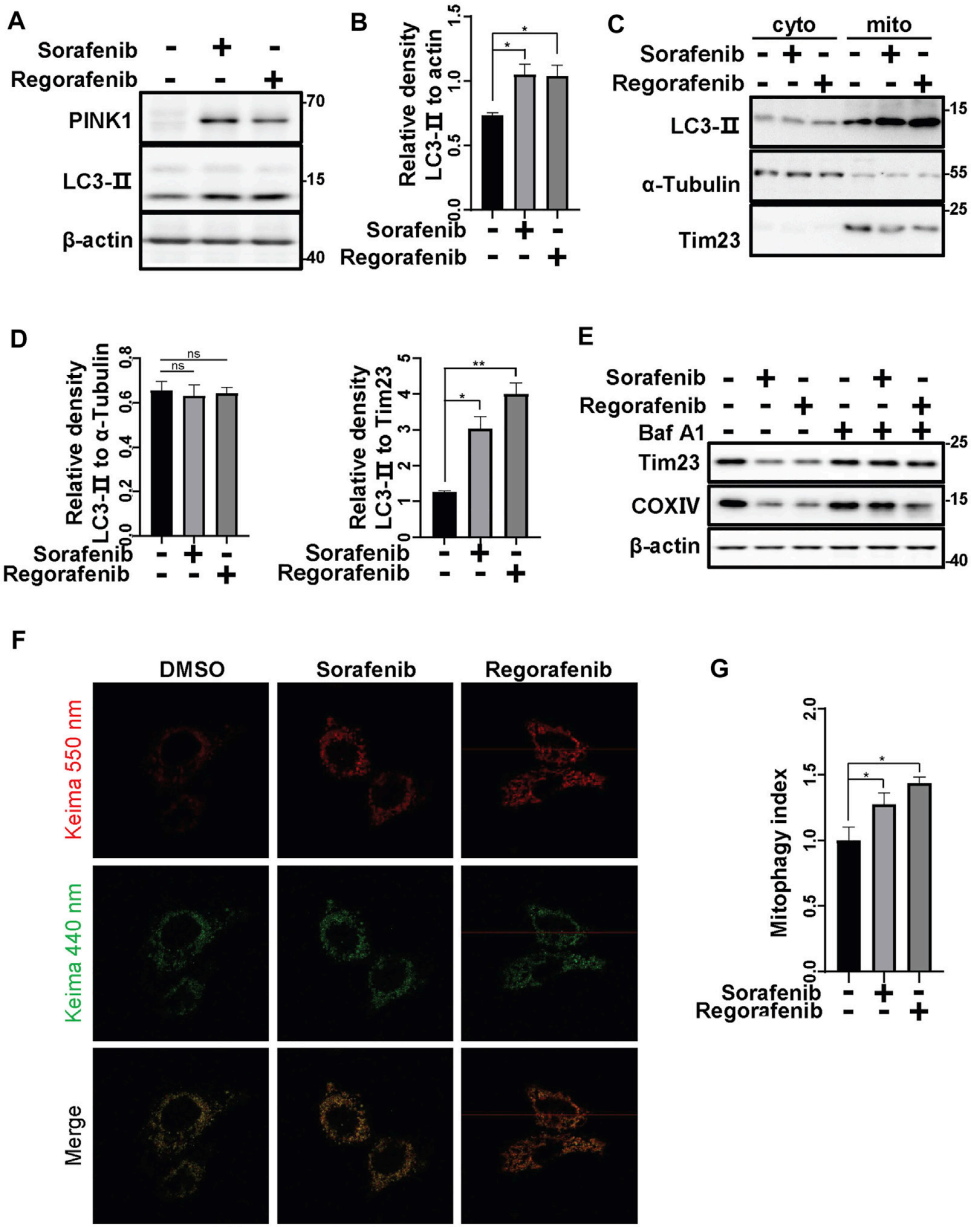
Sorafenib and regorafenib induce mitophagy. **(A)** HepG2 cells were treated with sorafenib (20 μM) or regorafenib (30 μM) for 12 h. LC3-II protein levels were detected using western blotting. **(B)** The relative densities of LC3-II were quantified, with the protein level normalized to the loading controls (β-actin). Values are the mean ± SEM from three independent experiments (*p < 0.05, t tests). **(C)** HepG2 cells were treated with sorafenib (20 μM) or regorafenib (30 μM) for 12 h. After treatment, the mitochondrial and cytosolic fractions were separated using a mitochondria isolation kit. LC3-II in the cytosol or mitochondria was labeled with the indicated antibodies. **(D)** The relative densities of LC3-II were quantified, with the protein level normalized to the loading control on mitochondria (Tim23) or in the cytosol (α-Tubulin). Values are the mean ± SEM from three independent experiments (*p < 0.05, **p < 0.01, one-way ANOVA and Tukey’s multiple comparisons test). **(E)** HepG2 cells were pretreated with Baf A1 (100 nM) for 1 h and then treated with sorafenib (20 μM) or regorafenib (30 μM) for 12 h. The Tim23 and COXIV proteins were labeled with the indicated antibodies. **(F)** HepG2 cells that were transfected with mito-keima for 24 h and then treated with sorafenib (20 μM) or regorafenib (30 μM) for 12 h were subjected to confocal microscopy imaging. **(G)** The relative fluorescence densities of the cells were quantified. Values are the mean ± SEM from three independent experiments (*p < 0.05, t tests).

As sorafenib and regorafenib decreased mitochondrial Tim23 upon the accumulation of PINK1, we further determined whether sorafenib and regorafenib can induce mitophagy. As LC3 conversion from LC3-I to LC3-II that anchors on the phagophores indicates an activation of autophagy (Wang and Wang, 2019a), we therefore examined the conversion of LC3. In HepG2 cells that were treated with sorafenib and regorafenib, LC3 conversion from LC3-I to LC3-II was increased (Figures 4A,B). Moreover, increases in LC3-II in mitochondrial fractions isolated from HepG2 cells that were treated with sorafenib and regorafenib were observed (Figures 4C,D), which indicates an increase in the recognition of mitochondria by autophagosomes. Meanwhile, the mitochondrial protein Tim23 was decreased in the mitochondrial fractions in cells that were treated with sorafenib and regorafenib (Figure 4C). As Tim23 is a mitochondrial inner membrane protein, the decrease of Tim23 suggests a degradation of mitochondria by mitophagy. In cells that were treated with bafilomycin A1, a lysosomal inhibitor, sorafenib- and regorafenib-induced decreases in the mitochondrial proteins Tim23 and COXIV were blocked (Figure 4E), indicating that bafilomycin A1 blocks the degradation of mitochondria. Thus, data suggest an induction of mitophagy by sorafenib and regorafenib. To further evaluate mitophagy, we used mitochondrial matrix-targeted Keima (MitoKeima), which is pH-sensitive. Upon mitophagy, the acidic environment of lysosomes shifts MitoKeima excitation from 440 to 550 nm. In untreated cells that expressed MitoKeima, a fluorescence signal at 440 nm was observed, but the fluorescence signal at 550 nm was weak (Figure 4F). However, in cells that were treated with sorafenib and regorafenib, the fluorescence signal at 550 nm was significantly increased (Figures 4F,G), further suggesting that sorafenib and regorafenib induce mitophagy.

### Inhibition of Mitochondrial Fission Increases the Cell Death Induced by Sorafenib and Regorafenib

**FIGURE 5:**
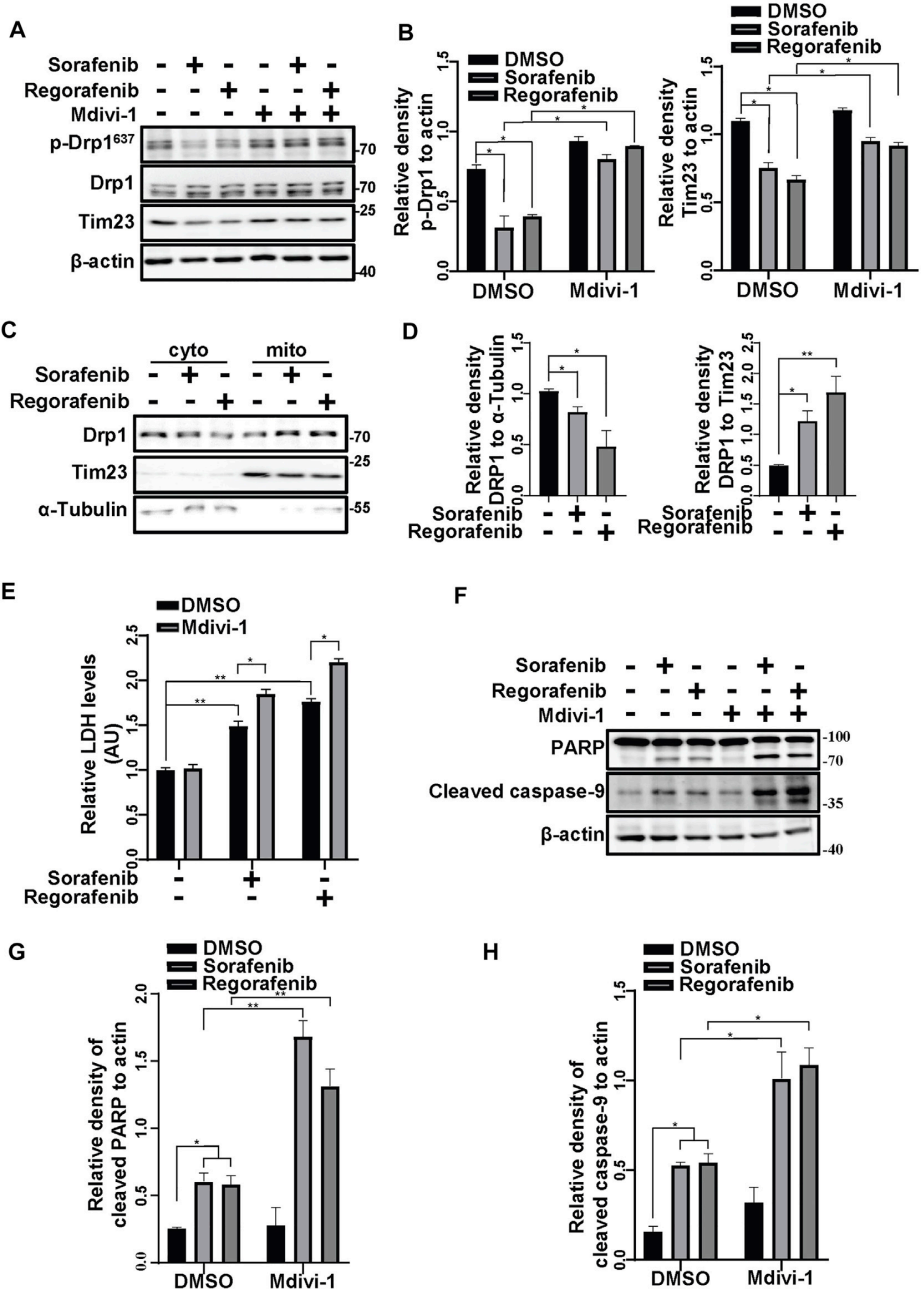
Inhibiting mitochondrial fission increases the cell death induced by sorafenib and regorafenib. **(A)** HepG2 cells that were pretreated with Mdivi-1 (10 μM) for 1 h and treated with sorafenib (20 μM) or regorafenib (30 μM) for 4 h were subjected to western blotting. p-Drp1, Drp1, and Tim23 were labeled with the indicated antibodies. **(B)** The relative densities of p-Drp1 and Tim23 were quantified, and the protein levels were normalized to the loading control (β-actin). Values are the mean ± SEM from three independent experiments (*p < 0.05, two-way ANOVA and Tukey’s multiple comparisons test). **(C)** HepG2 cells were treated with sorafenib (20 μM) or regorafenib (30 μM), and the mitochondrial and cytosolic fractions were separated using a mitochondria isolation kit. Drp1 protein levels in the cytosol or mitochondria were detected with immunoblot analysis. **(D)** The relative density of Drp1 was quantified, with the protein level normalized to the loading control on mitochondria (Tim23) or in the cytosol (α-Tubulin). Values are the mean ± SEM from three independent experiments (*p < 0.05, **p < 0.01, one-way ANOVA and Tukey’s multiple comparisons test). **(E)** Cytotoxicity was measured using LDH assays in HepG2 cells that were pretreated with mdivi-1 (10 μM) for 2 h and treated with sorafenib (20 μM) or regorafenib (30 μM) for 24 h. Values are the mean ± SEM from three independent experiments (*p < 0.05, **p < 0.01, two-way ANOVA and Tukey’s multiple comparisons test). **(F)** Cleaved PARP and cleaved caspase-9 in HepG2 cells that were treated as in **(E)** are shown**. (G,H)** The relative densities of cleaved PARP and cleaved caspase-9 were quantified, and the protein levels were normalized to the loading control (β-actin). Values are the mean ± SEM from three independent experiments (*p < 0.05, **p < 0.01, two-way ANOVA and Tukey’s multiple comparisons test).

The damaged mitochondrial segments are segregated by fission before they are engulfed by phagophores. Hence, we assessed the activation of Drp1, a protein that provides mitochondrial fission force. The phosphorylation of Drp1 at Ser637 negatively regulates Drp1 GTPase activity (Chang and Blackstone, 2007). In cells that were treated with sorafenib and regorafenib, Drp1 phosphorylation at Ser637 was decreased, accompanied by decreases in Tim23 levels (Figures 5A,B). Meanwhile, mdivi-1, a mitochondrial fission inhibitor that targets Drp1, restored the phosphorylation of Drp1 at Ser637 and the protein levels of Tim23 (Figures 5A,B). Moreover, using fractionation assays, we observed that sorafenib and regorafenib increased Drp1 translocation to mitochondria from the cytosol (Figures 5C,D), suggesting that sorafenib and regorafenib activate Drp1 and induce Drp1 mitochondrial translocation for mitochondrial fission and mitophagy. In HepG2 cells that were pretreated with mdivi-1, sorafenib and regorafenib induced more cell death than those without mdivi-1 treatment, which was evidenced by LDH release assays (Figure 5E). Moreover, the cleavage of PARP and caspase-9 induced by sorafenib and regorafenib was also increased upon mdivi-1 treatment (Figures 5F–H).

### Inhibition of Mitophagy Increases the Cell Death Induced by Sorafenib and Regorafenib *In Vitro* and *In Vivo*

**FIGURE 6:**
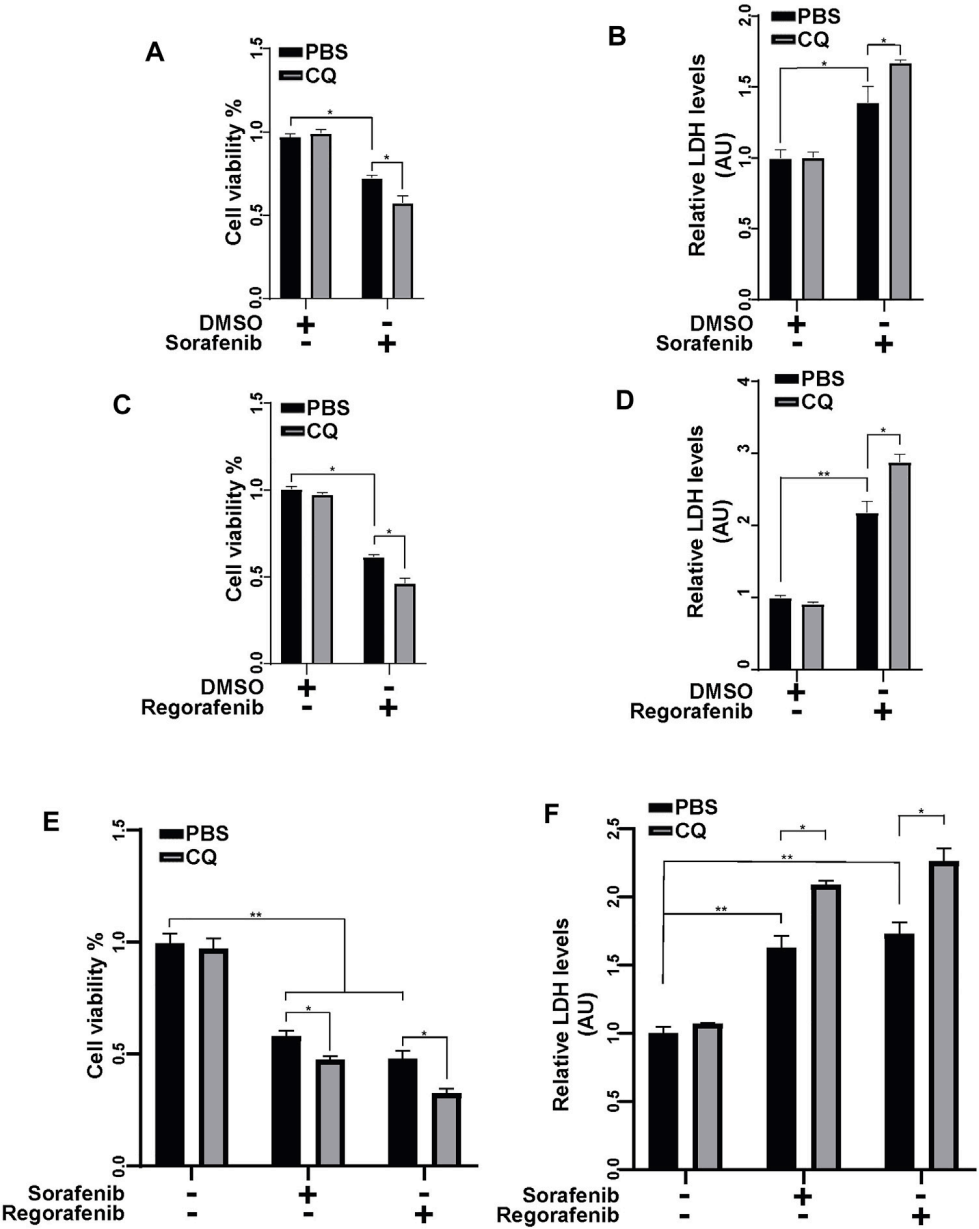
Inhibiting mitophagy enhances the anticancer effect of sorafenib and regorafenib in HCC cells. **(A)** Cell viability was measured using a CCK-8 kit in HepG2 cells that were pretreated with CQ (50 μM) for 2 h and then treated with sorafenib (20 μM) for 24 h. Values are the mean ± SEM from four independent experiments (*p < 0.05, two-way ANOVA and Tukey’s multiple comparisons test). **(B)** Cytotoxicity was measured using LDH assays in HepG2 cells that were treated as in A. Values are the mean ± SEM from three independent experiments (*p < 0.05, **p < 0.01, two-way ANOVA and Tukey’s multiple comparisons test). **(C,D)** Cell viability and cytotoxicity were measured in HepG2 cells that were treated as described in **(A,B)** but with regorafenib (30 μM). Values are the mean ± SEM from four independent experiments (*p < 0.05, **p < 0.01, two-way ANOVA and Tukey’s multiple comparisons test). **(E)** Cell viability was measured using a CCK-8 kit in Hep3B cells that were pretreated with CQ (50 μM) for 2 h and then treated with sorafenib (20 μM) or regorafenib (30 μM) for 24 h. Values are the mean ± SEM from four independent experiments (*p < 0.05, two-way ANOVA and Tukey’s multiple comparisons test). **(F)** Cytotoxicity was measured using LDH assays in Hep3B cells that were treated as in **(E)**. Values are the mean ± SEM from three independent experiments (*p < 0.05, **p < 0.01, two-way ANOVA and Tukey’s multiple comparisons test).

We showed that sorafenib and regorafenib induced mPTP opening and PINK1-mediated mitophagy. As the clearance of damaged mitochondria by mitophagy protects cells from mitochondria-induced cell death, we wondered whether sorafenib- and regorafenib-induced mitophagy has effects on cell survival. Inhibition of autophagy with CQ aggravated sorafenib- and regorafenib-induced cell death in HepG2 cells, which was detected using CCK-8 assays (Figures 6A,B) and LDH assays (Figures 6C,D). Similar results were obtained using Hep3B cells, showing that an inhibition of autophagy with CQ aggravated sorafenib- and regorafenib-induced cell death (Figures 6E,F).

**FIGURE 7:**
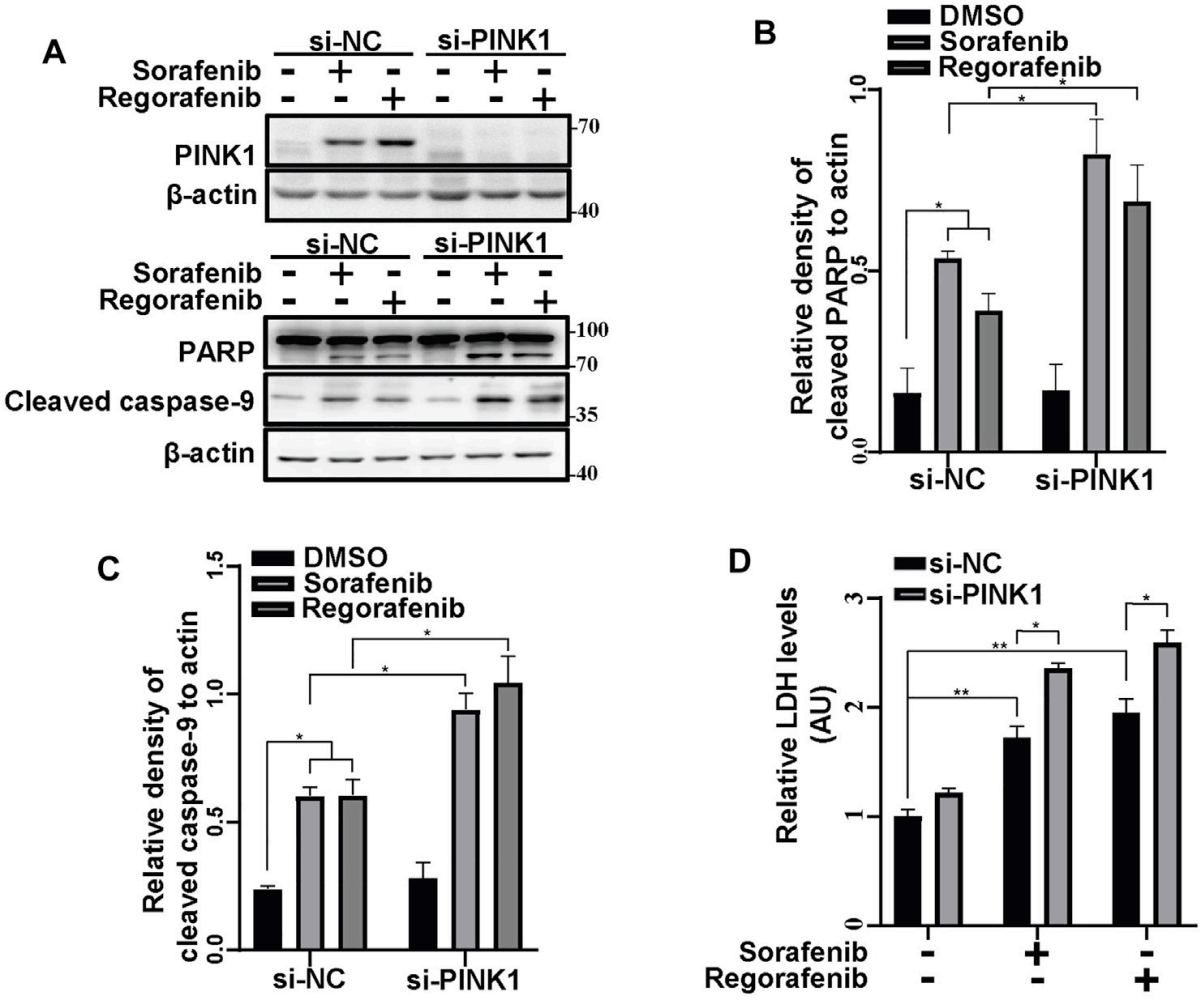
Inhibiting mitophagy by knockdown of PINK1 enhances the sorafenib- and regorafenib-induced cleavages of PARP and cleaved caspase-9. **(A)** PINK1, cleaved PARP and cleaved caspase-9 in HepG2 cells that were transiently transfected with siRNA to PINK1 for 48 and then treated with sorafenib (20 μM) or regorafenib (30 μM) for 24 h were labeled with the indicated antibodies. **(B,C)** The relative densities of cleaved PARP **(B)** and cleaved caspase-9 **(C)** were quantified, with the protein level normalized to the loading control (β-actin). Values are the mean ± SEM from three independent experiments (*p < 0.05, two-way ANOVA and Tukey’s multiple comparisons test). **(D)** Cytotoxicity was measured using LDH assays in HepG2 cells that were treated as in **(A)**. Values are the mean ± SEM from three independent experiments (*p < 0.05, **p < 0.01, two-way ANOVA and Tukey’s multiple comparisons test).

To further identify the effects of PINK1-mediated mitophagy on sorafenib- and regorafenib-induced cell death, we knocked down PINK1 in HepG2 cells and treated cells with sorafenib and regorafenib (Figure 7A). With sorafenib or regorafenib treatment, cleaved PARP and cleaved caspase-9 levels were both increased in PINK1 knockdown cells compared with those in PINK1 wild-type cells (Figures 7A–C). LDH assays showed that sorafenib and regorafenib induced more cell death in cells in which PINK1 was knocked down (Figure 7D).

**FIGURE 8:**
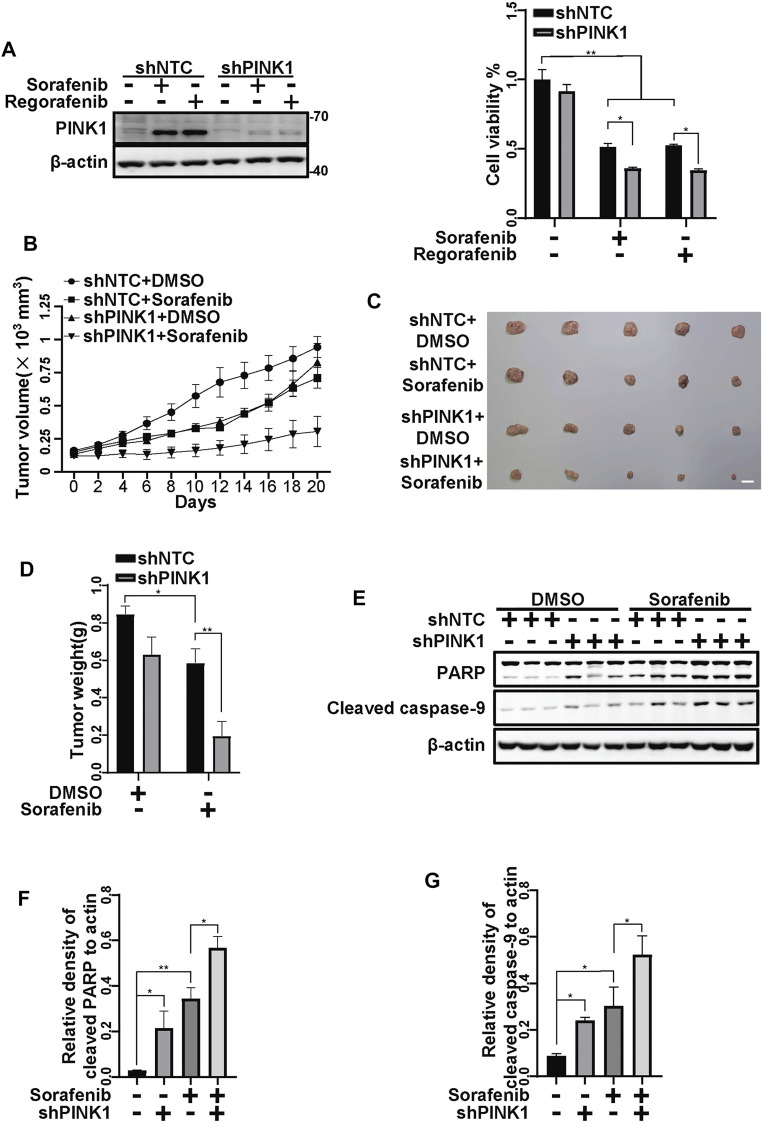
Targeting PINK1 effectively promotes sorafenib repressive effects on tumor growth *in vivo*. **(A)** HepG2 cells that stably expressed shNTC or shPINK1 were treated with sorafenib (20 μM) or regorafenib (30 μM) for 24 h and subjected to western blotting with the indicated antibodies. Cell viability was measured using CCK-8 kit. Values are the mean ± SEM from four independent experiments (*p < 0.05, **p < 0.01, two-way ANOVA and Tukey’s multiple comparisons test). **(B)** Growth curves of tumors that stably expressed shNTCs or shPINK1 in animals treated with or without sorafenib are shown. **(C,D)** Representative xenograft tumors at the endpoint are shown. The graph shows the weight of tumors in each group (*n* = 5, scale bar, 1 cm *p < 0.05, **p < 0.01, two-way ANOVA and Tukey’s multiple comparisons test). **(E)** The cleavages of PARP and caspase-9 in tumor tissues from **(B)** were detected using immunoblot analysis. **(F,G)** The relative densities of cleaved PARP **(F)** and cleaved caspase-9 **(G)** were quantified, and the protein levels were normalized to the loading control (β-actin). Values are the mean ± SEM from three independent experiments (*p < 0.05, **p < 0.01, two-way ANOVA and Tukey’s multiple comparisons test).

**FIGURE 9:**
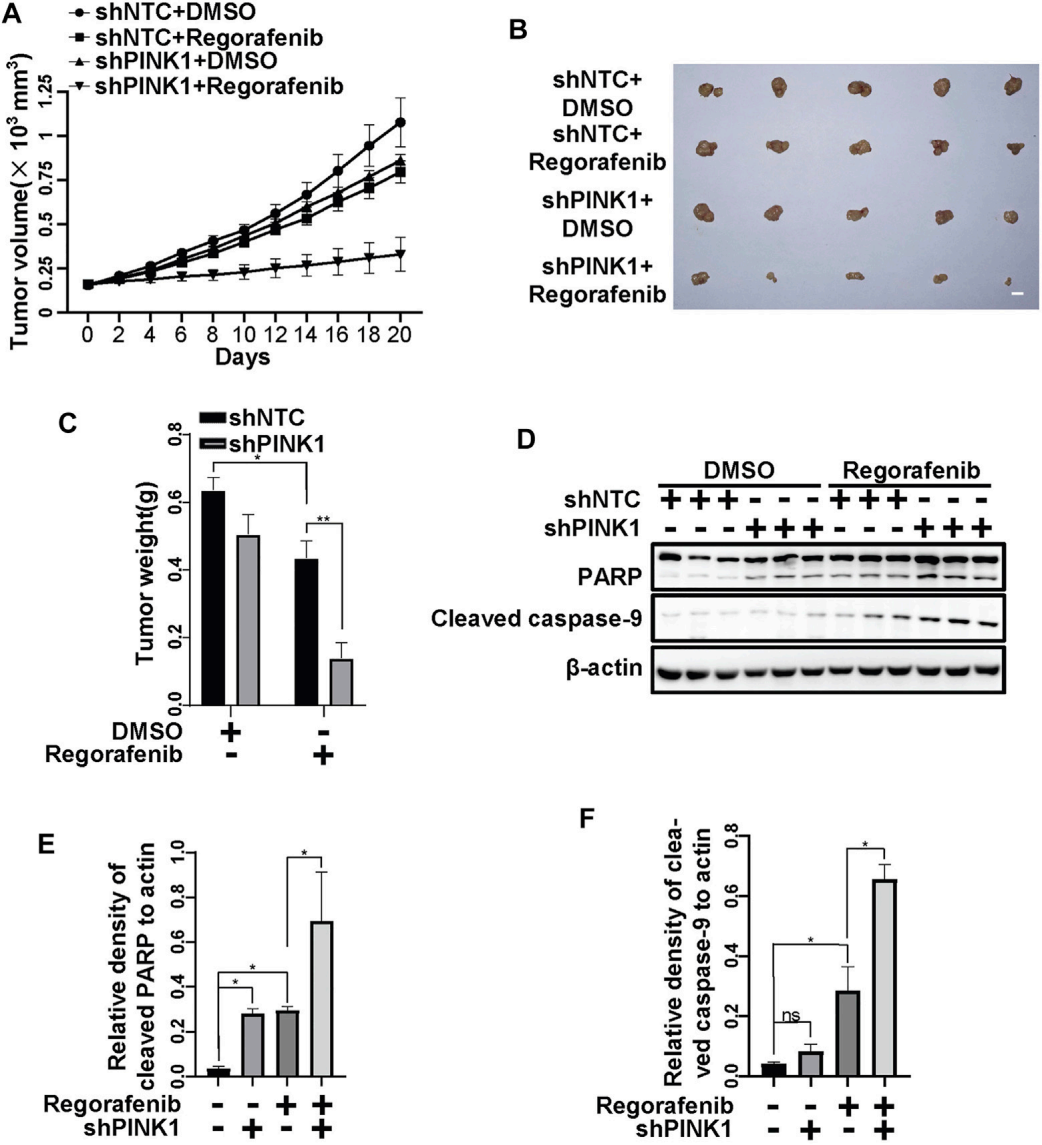
Targeting PINK1 effectively promotes regorafenib repressive effects on tumor growth *in vivo*. **(A)** Growth curves of tumors that stably expressed shNTCs or shPINK1 in animals treated with or without regorafenib are shown. **(B)** Representative xenograft tumors at the endpoint are shown. **(C)** The graph shows the weight of tumors in each group (*n* = 5, scale bar, 1 cm *p < 0.05, **p < 0.01, two-way ANOVA and Tukey’s multiple comparisons test). **(D)** The cleavages of PARP and caspase-9 in tumor tissue from **(A)** were detected using immunoblot analysis. **(E,F)** The relative densities of cleaved PARP **(E)** and cleaved caspase-9 **(F)** were quantified, and the protein levels were normalized to the loading control (β-actin). Values are the mean ± SEM from three independent experiments (*p < 0.05, two-way ANOVA and Tukey’s multiple comparisons test).

To further examine the protective role of PINK1, we constructed HepG2 cells stably shPINK1 in which PINK1 was knocked down. Consistent with our data from transient PINK1 knockdown cells, sorafenib and regorafenib treatment resulted in a greater decrease in cell viability in shPINK1 HepG2 cells than in shNTC cells (Figure 8A). We next established a mouse xenograft model using shNTC or shPINK1 HepG2 cells. Compared with the control groups, PINK1 knockdown or sorafenib treatment alone significantly delayed tumor growth (Figures 8B,C). Moreover, the growth of tumors in nude mice that were treated with sorafenib was much slower in those harboring shPINK1 than in those harboring shNTC (Figures 8B,C). Furthermore, the tumor weight in nude mice that were treated with sorafenib was much lower in those harboring shPINK1 than in those harboring shNTC (Figures 8C,D). Biochemical analyses also showed that there was more cleavage of PARP and caspase-9 in tumors harboring shPINK1 than in those harboring shNTC in nude mice that were treated with sorafenib (Figures 8E–G). Similar results were obtained in mice that were treated with regorafenib. PINK1 knockdown or regorafenib treatment alone significantly delayed tumor growth (Figures 9A–C). The growth of tumors in nude mice that were treated with regorafenib was slower in those harboring shPINK1 than in those harboring shNTC (Figures 9A–C). Furthermore, the tumor weight in nude mice that were treated with regorafenib was much lower in those harboring shPINK1 than in those harboring shNTC (Figures 9B,C). In addition, more cleavage of PARP (Figures 9D,E) and caspase-9 (Figures 9D,F) was observed in tumors harboring shPINK1 than in those harboring shNTC in nude mice that were treated with regorafenib.

## Discussion

HCC is one of the most common fatal malignancies and a major cause of cancer-related deaths due to a lack of effective therapies. As patients with advanced HCC are not eligible for surgical treatments, sorafenib is used as the first-line therapy for patients with HCC, although most patients eventually gain disease progression. Regorafenib is a second-line treatment for patients with HCC progression on sorafenib. Here, we demonstrate that sorafenib and regorafenib induce mPTP opening by mtCa^2+^ overload. On the one hand, sorafenib and regorafenib cause mitochondria-related cell death. On the other hand, they activate PINK1/Parkin-regulated mitophagy, which hinders mitochondria-induced tumor-killing activity.

mPTP is a CsA-sensitive high conductance channel on mitochondria. It has been reported that the mPTP consists of several components, including the outer membrane voltage-dependent anion channel (VDAC), adenine nucleotide translocase (ANT), cyclophilin D (CypD) and ATP synthase. Transient openings are physiological and maintain cellular homeostasis (Huser and Blatter, 1999); however, prolonged opening leads to the outflow of respiratory substrates and the swelling of mitochondria, which induce necrosis and apoptosis (Petronilli et al., 2001; Vaseva et al., 2012; Izzo et al., 2016). A large number of compounds have been identified for their chemotherapeutics by eliciting mPTP opening in different pathways. BAY-2234 induces cell death in melanoma cells through ROS-activated mPTP opening (Basit et al., 2017). Hirsutine causes lung cancer cell death by GSK3β-mediated mPTP opening (Zhang et al., 2018). Detaching hexokinase II using a selective peptide can also induce mPTP opening and apoptosis in several cancer cells (Chiara et al., 2008). Here, we found that sorafenib and regorafenib induce mPTP opening, resulting in cell death in HCCs. Blockage of mPTP by CsA alleviates sorafenib- and regorafenib-induced cell death. mtCa^2+^ overload is one of the major causes of mPTP opening (Kwong, 2017). The opening of the mPTP can cause mitochondrial depolarization and ROS production (Kent et al., 2021). In our observations, sorafenib and regorafenib increased mtCa^2+^ overload, which was evidenced by the calcium indicator mito-pericam. Furthermore, using the calcium chelating agent BAPTA partially restored the ΔΨm. Thus, our data suggest that sorafenib- and regorafenib-induced mtCa^2+^ overload and mPTP opening contribute to the collapse of ΔΨm and mitochondria-mediated cell death.

Mitophagy is a cellular process that clears damaged mitochondria (Wang Y. et al., 2019). Once mitophagy is activated, damaged mitochondria will be recognized by different autophagic receptor (p62/SQSTM1, OPTN, FUNDC1, and NIX) that bind to LC3, leading to an engulfment of the damaged mitochondria by the phagophores for autophagic degradation (Wang and Wang, 2019b). It has been documented that the induction of mitophagy by anticancer treatments counteracts drug-induced mitochondrial damage and cytotoxicity. Inhibition of mitophagy increases the cancer cell death induced by chemotherapy (Abdrakhmanov et al., 2019). Knockdown of key mitophagy regulators, such as PINK1, FUNDC1 or AMBRA1, also improves the efficiency of anticancer treatment (MacKeigan et al., 2005; Hou et al., 2017; Liu et al., 2019). Inhibiting NIX-mediated mitophagy increases the sensitivity to doxorubicin in cancer stem cells (Yan et al., 2017). These reports suggest a prosurvival role of mitophagy in cancer cells during anticancer treatments. Upon the collapse of ΔΨm, PINK1 is accumulated on mitochondria and recruits Parkin to mitochondria (Jin et al., 2010; Matsuda et al., 2010; Gao et al., 2020). The mitochondria that are ubiquitinated by Parkin can be recognized and engulfed by phagophores to form autophagosomes that are fused with lysosomes, leading to degradation of mitochondria (Narendra et al., 2010; Youle and Narendra, 2011; Gao et al., 2015). In the present study, we showed that PINK1 accumulates on mitochondria and recruits Parkin to mitochondria in cells in which the ΔΨm are collapsed after sorafenib or regorafenib treatment. CsA treatment that restores the ΔΨm damaged by sorafenib or regorafenib also blocks PINK1 accumulation. PINK1-mediated clearance of mitochondria that are damaged by sorafenib or regorafenib treatment has protective effects against cell death, as knockdown of PINK1 aggravates the cell death induced by sorafenib and regorafenib. Moreover, the average volume of tumor xenografts with shPINK1 was smaller than that with shNTC, further suggesting that PINK1-mediated mitophagy alleviates sorafenib- or regorafenib-induced antitumor effects.

Before mitophagy, the dysfunctional mitochondrion is segregated to two smaller mitochondria by mitochondrial fission: one is healthy, and the other is depolarized (Dorn and Kitsis, 2015). The healthy mitochondria will be fused with other healthy mitochondria to perform normal functions. The damaged one will be engulfed by autophagosomes for degradation. Drp1 is the key regulator during mitochondrial fission. Mitochondrial fission has been reported to promote the survival of a number of cancer cells, including HCC. Under hypoxic conditions, HCC cells activate mitophagy by upregulating Drp1 expression and its activity to induce mitochondrial fragmentation (Lin et al., 2020). Furthermore, increased mitochondrial fission is related to poor prognosis in HCC patients (Huang et al., 2016). The phosphorylation of Drp1 is the main posttranslational modification that regulates Drp1 activity. Phosphorylation of Drp1 at S637 inhibits its activity (Chang and Blackstone, 2007). Here, we showed that sorafenib and regorafenib induced the activation and translocation of Drp1 to mitochondria. Inhibition of Drp1 with mdivi-1 accelerated the cell death induced by sorafenib and regorafenib.

**FIGURE 10:**
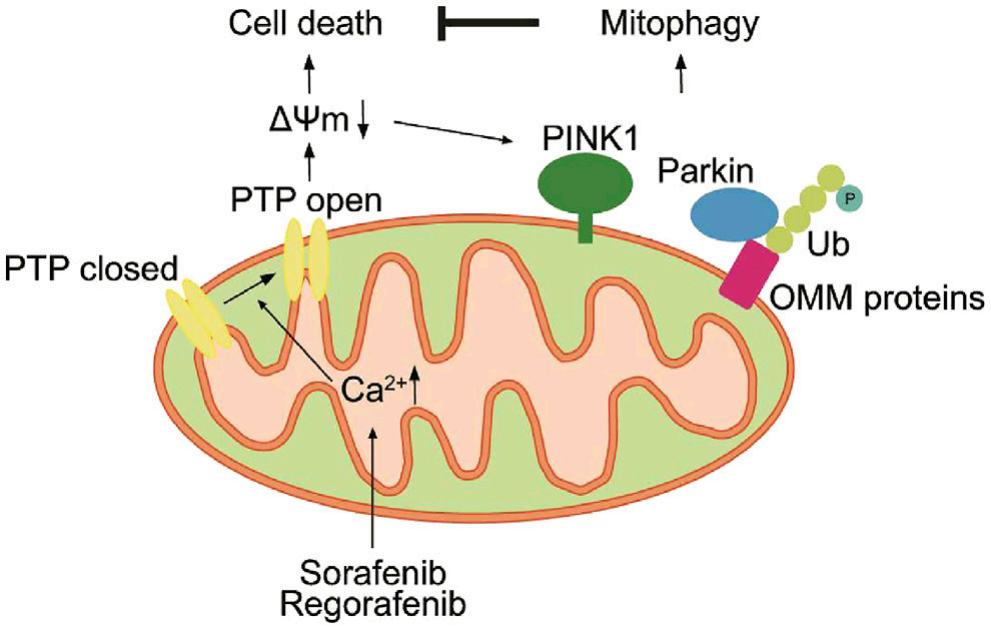
Sorafenib and regorafenib induce mPTP opening due to mtCa^2+^ overload. The opening of the mPTP induces cell death and the collapse of ΔΨm. However, loss of ΔΨm activates PINK1-mediated mitophagy, which in turn alleviates sorafenib- and regorafenib-induced cell death.

In summary, we demonstrate that sorafenib and regorafenib induce mPTP opening due to mtCa^2+^ overload. The opening of the mPTP induces cell death; however, it also induces the collapse of ΔΨm, which activates PINK1-mediated mitophagy. PINK1-mediated mitophagy alleviates sorafenib- and regorafenib-induced cell death both *in vitro* and *in vivo* (Figure 10). Thus, our study suggests that targeting PINK1 or blocking mitophagy are potential therapeutic strategies in sorafenib and regorafenib treatment.

## Data Availability

The original contributions presented in the study are included in the article/supplementary material, further inquiries can be directed to the corresponding authors.
